# Effects of Superimposed Blood Flow Restriction on Isokinetic Knee Extension

**DOI:** 10.3390/jfmk10020167

**Published:** 2025-05-10

**Authors:** Darío Rodrigo-Mallorca, Joaquín Mollá-Sanchis, Iván Chulvi-Medrano, Luis M. Franco-Grau

**Affiliations:** 1 Department of Physical and Sport Education, University of Valencia, 46010 Valencia, Spain; mallorca@alumni.uv.es (D.R.-M.); ximokm42@gmail.com (J.M.-S.); luis.m.franco@uv.es (L.M.F.-G.); 2 Research Group in Prevention and Health in Exercise and Sport (PHES), Department of Physical and Sport Education, University of Valencia, 46010 Valencia, Spain

**Keywords:** resistance training, vascular occlusion, acute responses, muscle strength

## Abstract

**Objective:** To evaluate changes in the isokinetic concentric moment of the knee extensors and the moment–velocity curve during the application of no BFR compared to superimposed BFR. **Methods:** A total of 37 physically active adults [33.73 (10.96) years; 11 females] performed three sets of isokinetic concentric knee extensions, each including three angular velocities (300°/s, 210°/s, and 120°/s; BIODEX dynamometer). BFR at 40% (BFR40) and 80% (BFR80) of the maximal pressure occlusion (MPO) were applied randomly after an equal control protocol without BFR (BFR0). **Results:** No significant differences were found for any interaction between the BFR condition and angular velocity (*p* > 0.05); 109.78 ± 32.90 vs. 71.24 ± 11.18, 116.68 ± 27.29 vs. 74.40 ± 15.11, and 113.91 ± 28.43 vs. 72.95 ± 13.76 Nm at 300°/s; 137.60 ± 35.27 vs. 88.85 ± 15.23, 135.40 ± 33.04 vs. 86.32 ± 17.38, and 132.68 ± 31.99 vs. 85.39 ± 16.25 Nm at 210°/s; 177.62 ± 41.40 vs. 114.72 ± 20.10, 166.40 ± 45.39 vs. 198.14 ± 21.80, and 162.60 ± 40.10 vs. 109.09 ± 18.90 Nm at 120°/s, for BFR0, BFR40, and BFR80, respectively. There were significant differences in the interactions by gender. **Conclusions:** Superimposed application of BFR at 40% MPO and 80% MPO during an isokinetic knee extension did not cause any acute change in the ability to produce maximal moment or power. The use of BFR may not generate an ergogenic effect that is sufficient to cause acute changes in force production.

## 1. Introduction

Combining low-intensity resistance training [20–30% of One Repetition Maximum (1RM)] with blood flow restriction (BFR) has been shown to induce physiological adaptations resulting in improvements of strength and muscle mass at a similar extent to those obtained by traditional resistance training with higher loads (>75% 1RM) [1,2,3,4]. BFR training consists of the use of a tourniquet made by an inflatable cuff placed on the proximal part of the limb (e.g., arm or leg), generating an external high-pressure level that partially reduces arterial blood flow during exercise and impairs venous return [5].

Among others, the mechanisms favoring the chronic positive effects of BFR training on strength and muscle mass are related to physiological responses, such as increased mechanical strain, metabolic stress [6], cellular inflammation, and recruitment of fast twitch type II muscle fibers [7]. Regarding the acute effects derived from the use of BFR, Wilk et al. [8,9] theorized that the mechanical work generated by the pressure of the inflatable cuff could potentially increase the speed of execution in resistance exercises such as the bench press. These authors suggest that, despite being a passive element, it appears that the tension and deformation of the material cuff can store and dissipate additional elastic energy, possibly resulting in improved power throughout the BFR contraction compared to no BFR.

Going further in the acute responses of BFR, Wilk et al. reported [8,9] that there is little knowledge of the influence of BFR on the rate of force development (RFD) and the acute changes in the force–velocity curve compared to no BFR, with most of the available data suggesting no acute physiological effects of BFR on neuromuscular capabilities [9]. In addition, most of the published studies have been conducted with linear encoders, while, to the best of our knowledge, no studies have analyzed muscular adaptations to BFR training using isokinetic devices, despite the fact that they represent the gold standard method for assessing maximal voluntary contraction (MVC) [10]. Because isokinetic devices allow the measurement of the MVC at a constant speed in a reliable and reproducible manner along the entire range of motion, an isokinetic graded test, including different angular velocities, would help to precisely determine any influence of BFR on the MVC and the power production with respect to different loads [10]. Moreover, accounting for the disparity of the methodological recommendations regarding the percentage of MOP in the inflatable cuff, it is also interesting to compare different pressures to elucidate any influence related to the mechanical effect of the cuff, for example, 40 and 80% of MPO, the minimum and maximum recommended values for exercise protocols incorporating BFR [5].

Therefore, determining whether there is any change in MVC, RFD, or power compared to no BFR, and/or any change in the force–velocity curve following a graded test, needs further attention and could be explored by means of an isokinetic assessment. The use of isokinetic devices, which are considered the gold standard for assessing maximal voluntary contraction (MVC), offers a unique opportunity to evaluate these acute responses with high precision, offering a unique advantage: it allows the precise measurement of muscle strength at any point within the range of motion. Little research has been conducted on the effects of BFR on the isokinetic variables. Previous research has examined the physiological responses of load-matched isokinetic versus isotonic BFR resistance exercises [11,12,13], showing contradictory results that could be attributed to the different acute responses of the isokinetic stimulus supplemented with BFR.

Thus, the main objective of the present study was to evaluate changes in the ability to generate maximal force in knee extensions at three different angular velocities (300°/s, 210°/s, and 120°/s) using an isokinetic device during the application of no BFR compared with superimposed BFR at 40% and 80% of maximum occlusion pressure (MPO). We hypothesized that the isokinetic graded test would confirm the absence of BFR influence in the acute ability to generate force irrespective of the load and/or cuff pressure, according to the scarce previous literature where no acute neural effects have been described.

## 2. Materials and Methods

### 2.1. Participants

**Figure 1:**
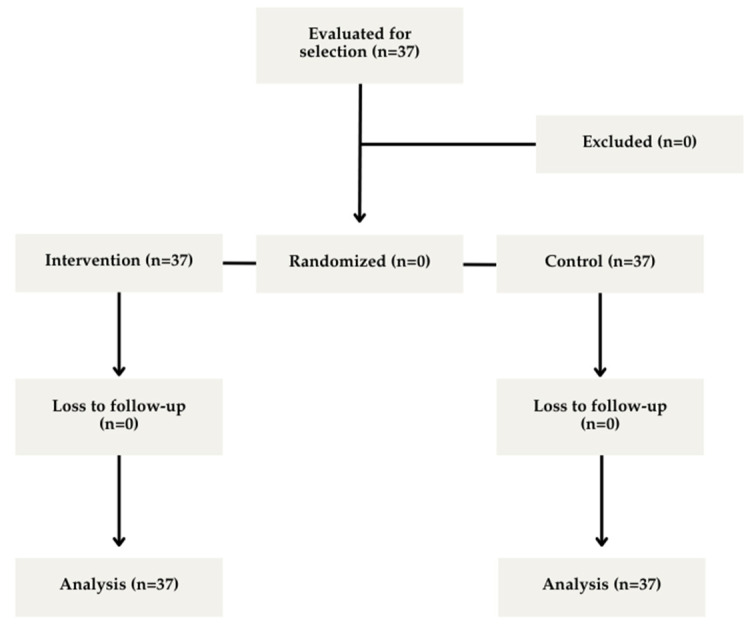
Flow chart of process of study.

Thirty-seven healthy adult subjects; 26 men [34.04 (11.26) years; 177.65 (0.07) cm; 70.61 (7.33) kg; 22.70 (2.79) kg/m^2^] and 11 women [33.00 (10.70) years; 162.29 (0.05) cm; 54.18 (6.56) kg; 20.73 (2.24) kg/m^2^] volunteered to participate in this study (Figure 1). A sample size calculation was previously carried out using the G*Power tool version 3.1.9.7 (total sample size: 21; effect size f: 0.8; alpha error: 0.05; beta error: 0.95; critical F value: 3.55). Inclusion criteria were as follows: (A) healthy individuals aged between 18 and 50 years; (B) physically active with a minimum of one year of experience in resistance training, particularly in knee extension exercises; and (C) right leg dominant. Exclusion criteria were as follows: (A) not meeting at least one inclusion criterion; (B) contraindications for BFR training: prostheses in the area to be occluded, cardiovascular conditions, deep vein thrombosis, varicose veins, uncontrolled hypertension, and pregnancy; (C) presenting musculoskeletal injuries in the limb involved in this study, and/or (D) general contraindications for physical exercise.

Signed informed consent was obtained from all subjects prior to the experimental procedures, which were approved by the ethics committee of the University of Valencia (Number: 1569115). Investigations involving humans were conducted in accordance with the ethical standards of the 2013 updated Declaration of Helsinki.

### 2.2. Experimental Design

All tests were performed at the same facility and under the same environmental conditions. The intervention was performed in a single session, and repeated measurements were taken in a random manner. All tests were performed by the same investigator, that is, a specialist in conditioning and strength, with more than 1 year of experience in the technique of blood flow restriction and isokinetic dynamometry. Familiarization sessions were not included because of the participants’ prior training experience with BFR and the use of the isokinetic system. However, this decision acknowledges the potential limitations of controlling the initial variability in neuromuscular responses.

Initially, height and body composition measurements were taken using bioelectrical impedance scales (Tanita DC-430MA, Tokyo, Japan) to obtain all parameters and anthropometric characteristics of the participants. To determine the value of the maximal pressure occlusion (MPO), the subject was placed in the supine position with a passive knee flexion of approximately 20–30° (measured with a goniometer) and a slight external rotation of the hip. The ultrasound probe was placed transversally to the thigh, in the middle third of the inner thigh, above the hiatus of the adductors [14]. The Doppler function of the ultrasound machine (Sonosite II, Fujifilm, Tokio, Japan) was used. The standard pneumatic arterial pressure cuff (Riester Komprimeter; Riester, Jungingen, Germany) located in the proximal portion of the leg was progressively inflated until the auscultatory pulse of the femoral artery was interrupted; at that pressure level, the MPO was established [15]. The cuff dimensions were 57 cm long × 9 cm wide. The pressures applied in this study were then 40% and 80% of MPO, which are standardized as the minimum and maximum recommended values for exercise protocols incorporating BFR [5].

The same cuff was used for all experimental sessions. The cuff was positioned in the inguinal region to ensure effective application. The inflation to the target pressure was performed within 2–3 s using the integrated pressure system of the device, facilitating precise control. Once the desired pressure was reached, it was maintained throughout all the contraction phases. During the rest period, the cuff was fully deflated to allow for vascular reperfusion and tissue recovery.

After the pre-intervention measurements were taken, a standardized general warm-up consisting of 5 min of cycloergometer pedaling at 60% of maximum heart rate (HR_max_) was established by Tanaka’s formula (207 − 0.7 × age) [16]. The specific warm-up consisted of one set of 15 repetitions at a moderate execution speed at 210°/s of knee extension with an isokinetic machine (Biodex System 4 Pro, Biodex Medical Systems Inc., New York 11967 USA). The participants were tested in a seated position with the knee under testing flexed at 90°, adjusting the axis of the mechanical arm of the machine to the approximate height of the condyles (anatomical axis). The height of the seat and depth of the backrest were adjusted to place individuals in a precise position according to their height. Participants were then secured with restraining straps and harnesses to allow movement of the leg without compensatory movements of the body. Range of motion parameters, 90° flexion calibration, and weighting of the limb were conducted following the software requirements [17].

Before starting each individual isokinetic test, the experimental protocol previously assigned through a randomization process (Research Randomizer) was loaded into the software (Biodex System 4.42). The non-BFR condition was assigned as a control at the beginning of each test for every participant; however, the following sets under occlusion at 40% and 80% of the MPO were performed in a randomized and blinded manner.

The evaluation was related to the extensors of the dominant knee and consisted of three angular velocities: 300, 210, and 120°/s for each of the BFR conditions. Lower speeds were not used due to evident discomfort in the pilot sessions. The intention was to simulate an incremental protocol for maximal strength assessment, in which the loads increase linearly from light to maximum [18]. In isokinetic dynamometry, this load progression is achieved by decreasing the angular velocity [19]. For this purpose, five maximal repetitions of knee extensions were performed at three predefined velocities, with a 2 min rest between sets and the BFR condition to allow complete recovery. The same process was repeated in the two conditions of BFR (40% and 80% MPO) in a randomized manner. All the strength data obtained from the tests were measures of absolute strength.

### 2.3. Statistical Analysis

Data were registered in an Excel spreadsheet designed ad hoc and further migrated to IBM^®^ SPSS^®^ Statistics 26.0. The peak moment (PM, in Nm) and average power (AP, in watts) values were retained for further analysis. The main results are presented as the median and interquartile range, together with confidence intervals (95% CI).

To further understand the acute effect of the different BFR conditions on the constant speed of execution, the Shapiro–Wilk test was first performed to check the normality of data distribution (both for the overall sample and for males and females separately), followed by the homogeneity of variances test through Levene’s test. All analyzed variables were non-normally distributed; thus, the Friedman nonparametric test was used to analyze the effect of different BFR conditions on the constant speed of execution. To analyze the interaction of sex by speed and occlusion conditions, repeated measures ANOVA was performed. The data are presented as the median and interquartile range (IQR). The significance level was set at *p* < 0.05. Effect sizes were measured by Cohen’s d and interpreted as follows: *d* < 0.2 = null effect, *d* < 0.5 = small effect, *d* < 0.8 = medium effect, and *d* > 0.8 = large effect [20].

## 3. Results

The descriptive characteristics of the participants are summarized in Table 1.

Considering the sample, no significant differences were found for any interaction between the BFR condition and angular velocity (*p* > 0.05). The peak moment showed no significant differences between the BFR condition: 120.8 (136.19), 104.11 (31.05), and 101.73 (31.21) Nm at 300°/s; 123.11 (37.93), 120.81 (36.87), and 118.62 (35.56) Nm at 210°/s; and 158.92 (46.39), 175.83 (151.95), and 146.69 (42.79) Nm at 120°/s, for BFR0, BFR40, and BFR80, respectively.

Regarding the results related to the effect of BFR on force production at different velocities, Table 2 and Table 3 present the changes in PM and AP, as well as the mean power in the three BFR conditions (BFR0, BFR40, and BFR80), for the three constant velocities (300°, 210°, and 120°) in males and females, respectively. Again, no significant differences were found (*p* > 0.05) with a small effect size (*d* < 0.5).

The effect size was small for most comparisons between experimental conditions. The minimum value recorded was *d* = 0.01 in the mean power calculated between the 40% BFR and 80% BFR conditions at an isokinetic speed of 120°/s in males, while the maximum value recorded was *d* = 0.57 in the mean moment calculated between the 40% BFR and 80% BFR conditions at an isokinetic speed of 120°/s in females.

However, in the subsequent analysis of the interaction by sex, significant differences were found for both BP and MP (Table 4).

## 4. Discussion

Our aim was to evaluate changes in the ability to generate maximal force in knee extensions at different velocities using an isokinetic device during the application of no BFR and superimposed BFR at 40% and 80% MPO. The main finding of this study was that an overlapping BFR affected neither positively nor negatively neuromuscular abilities, with no significant differences in force development. The effect size was very low according to Rhea’s (2004) scales, with a maximum recorded value of *d* = 0.57 for the intersection between 0% BFR and 80% BFR conditions [20].

It is interesting to note that, in this study, we observed no changes, although it has been speculated that the BFR forces the fast motor units to engage from the first effort due to the hypoxic environment induced by the cuff [21]. Our results suggest that BFR does not acutely alter neuromuscular capacities such as moment or power production in isokinetic muscle contractions, even though it may be a suitable strategy to improve other variables related to neuromuscular performance such as sprinting or jumping markers of athletic performance [4] and muscle hypertrophy [3], according to previous studies. These findings point out the idea that the nature of the exercise, or the way that BFR is applied, influences the effects of this strength training strategy.

The absence of changes in the moment and power is consistent with the results of a previous study by Wilk et al [9]. We speculate that muscle contraction in our testing session was not hard enough to induce neuromuscular changes that affect the ability to generate strength. On the one hand, Wilk’s investigations used the squat and bench press, whereas the present study was performed in unilateral knee extensions [9]. Despite the advantage of producing the maximal muscle contraction in the isokinetic assessment [22], it is likely that the biomechanics of the knee extension reduce the rebound effect suggested by Wilk. On the other hand, this rebound effect has been related to the compression of the device, suggesting increased power for wide (10 cm) versus narrow (4 cm) cuffs (8); however, in the present study, a 9 cm wide cuff was used, so it is likely that the absence of neuromuscular changes/improvements is related to the exercise and the protocol selected. Nevertheless, all current research is focused on the chronic effects of low-intensity BFR training using isotonic machines or free weights [23,24,25], and there are no studies analyzing acute BFR responses, nor using isokinetic dynamometry, so it is far too complicated to compare our results with any previous study.

A sex analysis was performed to determine the possible influence of this condition on the response when generating isokinetic maximal force with BFR since the work of Freitas et al. (2020) indicates that there are sex differences in muscle swelling and myoelectric activity of the vastus lateralis quadriceps [26]. Based on the results obtained in the aforementioned study, the authors suggest considering sex as a potential confounding variable. Along these lines, our results indicate that neither in men nor in women did BFR influence the capacity to generate force for any of the variables studied (*p* > 0.05). The apparent differences in results between men and women are not due to the use of BFR but to differences in neuromuscular performance.

Previous studies pointing to increases in the force–velocity profile induced by BFR assume the premise of possible mechanical compression effects associated with the material components of the cuff, the level of inflation [27], and the cell swelling itself as the factors that would explain an improvement in the ability to generate maximal force at different velocities. This mechanism has also been suggested as the cause of slight increases in muscle performance when using compressive garments [26]. Notwithstanding, when performing the knee extension isokinetic contraction in non-fatigue conditions (as in our protocol), no BFR condition has been able to induce any acute change, so the rebound effect might be not enough to increase strength in a very short maximal contraction where muscle fibers are likely to recover in each repetition.

Another plausible explanation for the ergogenic effect in force–velocity profiles states that the hypoxic environment induced by BFR may force the recruitment of IIx motoneurons [21], but exposure to hypoxia in an acute session may be insufficient. This fact is a speculation since the participation of the different motor neurons has not been assessed in our study, so future studies should take this into consideration.

When interpreting the results and conclusions of the present study, it is convenient to point out some limitations. First, the sample was heterogeneous. Second, no familiarization session was performed since the subjects were required to have extensive experience in resistance training and to perform the movement under study, and therefore, perhaps the perceptions of performing the exercise with BFR could alter, to some degree, the ability to generate force. Finally, the fact of not having used EMG and lower speeds, close to 60°/s, may be a limitation in obtaining the expected results. This is because the participants reported discomfort from the combination of blood flow restriction and speeds lower than 120°/s. Finally, it should be noted that the isokinetic stimulus was five repetitions, which is very different from the usual methodology of strength training with BFR, which was assumed to be one set of thirty repetitions followed by two sets of fifteen repetitions.

It is recommended that future research should focus on making comparisons with a larger sample by age range and sex and also investigate the evaluation of possible acute changes and chronic adaptations of the application of BFR at different pressure percentages, in order to determine if there is an optimal pressure level for improvements in the production of resistance or if there is a methodology for the application of BFR that reports improvements in muscle strength. Additionally, future research should incorporate electromyography (EMG) to evaluate motor unit recruitment patterns and near-infrared spectroscopy (NIRS) to monitor muscle oxygenation and optimize protocol variables, such as cuff design and pressure. Gender differences in physiological responses to BFR warrant further investigation, including analysis of hormonal influences on muscle swelling and recruitment patterns, which should be considered in study designs. Future research could focus on examining the effects of more traditional BFR training protocols, which usually involve one set of 30 repetitions followed by three sets of 15. This approach is different from the isokinetic protocol used in this study, which consisted of just five maximal repetitions. Comparing these methods could provide valuable insights into how varying training volumes and repetition schemes impact the outcomes of BFR training on neuromuscular acute responses.

## 5. Conclusions

This study demonstrates that the use of blood flow restriction (BFR) at 40% and 80% of maximal occlusion pressure (MOP) has no immediate impact on maximal strength or power output during knee extension exercises at different velocities. No notable gender variations were observed in the response of men and women to BFR.

These results suggest that BFR protocols may not provide the necessary boost to significantly improve acute neuromuscular performance. However, analyzing muscle activity variables using electromyography could provide greater robustness to the analysis.

This underscores the importance of tailoring BFR protocols to specific training goals, especially in rehabilitation settings or in populations unable to perform high-load resistance training.

## Figures and Tables

**Table 1:** Characteristics of participants (*n* = 37).

**Men**				
**Characteristics**	**N**	**Mean**	**SD**	**CI (95%)**
Age (years)	26	29.71	6.62	27.04–32.38
Height (cm)	26	177.71	6.97	174.89–180.53
Weight (kg)	26	83.04	12.75	77.89–88.19
BMI (kg/m^2^)	26	26.13	3.59	24.68–27.58
% Fat Mass	26	11.63	7.32	8.67–14.59
% Lean Body Mass	26	67.89	6.87	65.11–70.67
**Women**				
Age (years)	11	28.33	7.45	23.33–33.33
Height (cm)	11	163.03	8.64	157.23–168.83
Weight (kg)	11	67.5	13.81	58.22–76.78
BMI (kg/m^2^)	11	25.25	2.39	23.64–26.86
% Fat Mass	11	17.08	2.69	15.27–18.89
% Lean Body Mass	11	47.85	11.08	40.41–55.29

Abbreviations: BMI, body mass index; cm, centimeters; kg, kilograms; kg/m^2^, kilograms per square meter; SD, standard deviation; and CI, confidence interval.

**Table 2:** Male isokinetic variables: peak moment (Nm), and average power (w).

	0% BFR	40% BFR			80% BFR				
°/s	Median (IQR)	Median (IQR)	*p*_0–40_	*d*_0–40_	Median (IQR)	*p*_0–80_	*d*_0–80_	*p*_40–80_	*d*_40–80_
Peak Moment (Nm)									
120	169.45 (55.07)	161.75 (56.45)	0.31	0.25	158.20 (40.90)	0.13	0.37	0.51	0.09
210	127.75 (37.22)	131.65 (31.17)	0.43	0.06	129.35 (30.00)	0.19	0.15	0.33	0.03
300	103.35 (36.90)	109.25 (29.92)	0.27	0.23	109.60 (32.52)	0.26	0.13	0.39	0.09
Average Power (w)									
120	196.80 (64.07)	193.00 (106.15)	0.09	0.35	188.50 (80.15)	0.35	0.41	0.18	0.01
210	236.25 (111.92)	243.00 (119.80)	0.16	0.03	236.95 (86.10)	0.21	0.15	0.21	0.13
300	192.28 (110.48)	250.35 (109.85)	0.19	0.39	248.90 (101.10)	0.27	0.37	0.29	0.02

Abbreviations: °/s, degrees per second; Nm, newtons-meter; W, watts; and IQR, interquartile range.

**Table 3:** Female isokinetic variables: peak moment (Nm) and average power (w).

	0% BFR	40% BFR			80% BFR				
°/s	Median (IQR)	Median (IQR)	*p*_0–40_	*d*_0–40_	Median (IQR)	*p*_0–80_	*d*_0–80_	*p*_40–80_	*d*_40–80_
Peak Moment (Nm)									
120	113.30 (25.45)	116.50 (32.00)	0.13	0.39	109.09 (21.45)	0.19	0.28	0.31	0.43
210	87.20 (14.45)	82.80 (15.90)	0.11	0.15	85.39 (13.95)	0.26	0.22	0.21	0.05
300	74.40 (16.80)	73.40 (14.35)	0.20	0.24	72.90 (10.30)	0.18	0.14	0.24	0.10
Average Power (w)									
120	143.60 (17.15)	136.60 (18.50)	0.23	0.19	127.80 (20.40)	0.26	0.36	0.28	0.17
210	153.00 (42.25)	147.80 (47.70)	0.21	0.45	147.40 (33.05)	0.10	0.16	0.19	0.31
300	149.50 (42.85)	159.10 (34.90)	0.27	0.23	156.90 (42.80)	0.16	0.09	0.11	0.14

Abbreviations: °/s, degrees per second; Nm, newtons-meter; W, watts; and IQR, interquartile range.

**Table 4:** Gender interaction in velocity and BFR conditions.

	F	*p*	Eta-Squared
Peak Moment	3.72	0.027	0.096
Average Power	2.91	0.029	0.077

## Data Availability

The data presented in this study are available upon request from the corresponding author due to being part of a larger study to achieve a PhD.
